# Auburn and Vicinity

**Published:** 1916-11

**Authors:** 


### AUBURN AND VICINITY

Dr. R. R. McCully, formerly of Union Springs, N. Y., has opened an office for general practice on Court Street, Auburn.

Dr. Chas. D. Reid, Jr., has removed to Syracuse, N. Y.

Dr. W. P. Conway has purchased the house recently vacated by Dr. W. H. Coe at 82 East Genesee Street.

Dr. R. C. Almy of 130 East Genesee Street has purchased the property at 92 East Genesee Street, formerly occupied by Dr. Thos. C. Sawyer. The place is now being remodeled and will be ready for occupancy shortly.

Dr. Thos. C. Sawyer has removed to 97 North Street.

Dr. Geo. C. Sincerbeaux of 93 Wall Street has recently returned from three months' post-graduate study in Paediatrics in Boston and will remove to 96 East Genesee Street. His present location will be occupied by Dr. M. K. Willoughby, formerly of Genoa, N. Y.

Dr. R. W. H. Rollings, a former practitioner of this city, now of New York City, was united in marriage October 10 to Miss Hazel May Quick of East Genesee Street, Auburn. Mrs. Rollings is a graduate of the Flower Hospital (N. Y. city) Training School for Nurses.

